# Deep learning for automated detection of *Drosophila suzukii*: potential for UAV‐based monitoring

**DOI:** 10.1002/ps.5845

**Published:** 2020-04-20

**Authors:** Peter PJ Roosjen, Benjamin Kellenberger, Lammert Kooistra, David R Green, Johannes Fahrentrapp

**Affiliations:** ^1^ Laboratory of Geo‐Information Science and Remote Sensing Wageningen University & Research Wageningen The Netherlands; ^2^ UAV/UAS Centre for Environmental Monitoring and Mapping University of Aberdeen Aberdeen UK; ^3^ Institute of Natural Resource Sciences Zurich University of Applied Sciences Winterthur Switzerland

**Keywords:** *Drosophila suzukii*, deep learning, object detection, unmanned aerial vehicle (UAV), integrated pest management (IPM)

## Abstract

**BACKGROUND:**

The fruit fly *Drosophila suzukii*, or spotted wing drosophila (SWD), is a serious pest worldwide, attacking many soft‐skinned fruits. An efficient monitoring system that identifies and counts SWD in crops and their surroundings is therefore essential for integrated pest management (IPM) strategies. Existing methods, such as catching flies in liquid bait traps and counting them manually, are costly, time‐consuming and labour‐intensive. To overcome these limitations, we studied insect trap monitoring using image‐based object detection with deep learning.

**RESULTS:**

Based on an image database with 4753 annotated SWD flies, we trained a ResNet‐18‐based deep convolutional neural network to detect and count SWD, including sex prediction and discrimination. The results show that SWD can be detected with an area under the precision recall curve (AUC) of 0.506 (female) and 0.603 (male) in digital images taken from a static position. For images collected using an unmanned aerial vehicle (UAV), the algorithm detected SWD individuals with an AUC of 0.086 (female) and 0.284 (male). The lower AUC for the aerial imagery was due to lower image quality caused by stabilisation manoeuvres of the UAV during image collection.

**CONCLUSION:**

Our results indicate that it is possible to monitor SWD using deep learning and object detection. Moreover, the results demonstrate the potential of UAVs to monitor insect traps, which could be valuable in the development of autonomous insect monitoring systems and IPM. © 2020 The Authors. *Pest Management Science* published by John Wiley & Sons Ltd on behalf of Society of Chemical Industry.

## Graphical abstract

We studied the use of deep learning for unmanned aerial vehicle (UAV)‐based detection of *Drosophila suzukii* in sticky traps. Our results show that there is potential for UAVs to perform this task.

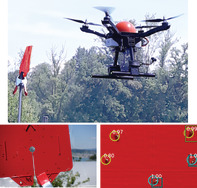

## 1 INTRODUCTION

Integrated pest management (IPM) aims to solve pest problems while minimising negative effects on the environment and human health.^1^ This includes minimising the use of pesticides by applying them to the right location, at the right time and in the right amounts. IPM seeks to continuously keep pests below the level at which they damage crops. An essential requirement for the successful implementation of IPM is the monitoring of pest populations.^2^ Accurate and timely knowledge of current pest populations provides information on when, where and which control measures are needed. Current methods for monitoring pest populations or pest presence typically involve field visits or are based on traps installed in an area of interest where target pest species are expected. Trap contents need to be analysed by an expert to identify the pest species caught. This process is costly, time‐ and labour‐intensive, and is prone to error.^3^ Moreover, repeatability, for example monitoring and thus visiting the same trap multiple times, or having to visit several traps at different locations over a large area, makes this a cumbersome and inefficient process.

Computer vision‐based monitoring of pests has the potential to overcome these issues by decreasing human effort and error, while increasing precision and speed.^4^ An expert in the field who has to inspect traps manually and classify their content could potentially be replaced by a camera system that monitors the traps. Detection of insects in traps would then rely on the localisation and recognition of insects in images of the traps taken by the camera system. Recently, computer vision‐based insect detection has received more attention due to increased popularity and advancements in the field of object detection and deep learning,^5^ especially with the use of convolutional neural networks (CNNs).^6^ Several studies have shown the potential of vision‐based insect monitoring. Zhong *et al*.^7^ developed a vision‐based counting and recognition system for six species of flying insects. In their approach, a camera was installed at a fixed and optimised position to monitor yellow sticky traps. In images collected by the camera, insects were detected and counted using the You Only Look Once (YOLO)^8^ algorithm and support vector machines (SVM). Similarly, Sun *et al*.^9^ developed a deep learning method for pest detection in cup traps based on images taken with a digital camera. In this system, the camera and an light‐emitting diode (LED) to control light exposure were placed at a fixed distance above the cup during image collection. By training a RetinaNet,^10^ Lin *et al*. they were able to distinguish their target pest (red turpentine beetle) from five other beetle species. Partel *et al*.^11^ adopted YOLO for the detection of citrus psyllids and Liu *et al*.^12^ trained several CNNs for the detection of different moth species in images captured in a camera‐trap.

In most studies using computer vision‐based approaches for insect detection, images were acquired under controlled viewing and/or illumination conditions to assure consistent image quality. Moreover, these approaches typically make use of imagery collected from a static viewpoint. A camera monitoring system based on such principals is non‐flexible and would require someone to physically visit each trap and collect images manually or set up a permanent camera in front of each trap. Unmanned aerial vehicles (UAVs), however, could fly from trap to trap while taking images. Several studies have been performed in which deep learning and UAV‐acquired images were used for detection tasks. For example, Kellenberger *et al*.^13^ demonstrated the possibility of detecting animals in wildlife reserves using deep learning and UAV imagery. Rivas *et al*.^14^ trained a CNN for detection of cattle in images collected by a UAV. However, UAV‐based detection of insects has not received much attention.^15^ Typically, the focus has been on detecting the effects of pests, such as crop damage, rather than the pests themselves.^16^, ^17^ The latter, is, however, more crucial for timely treatment and offers the possibility of preventing damage to crops.

In this study, we focus on computer vision‐based detection of pests. We investigated: (i) the possibility of using deep learning for vision‐based monitoring of insect traps under field conditions; and (ii) the potential of insect detection in images collected by UAVs. We specifically focused on the detection of *Drosophila suzukii* (Matsumura) (Diptera: Drosophilidae), commonly known as the spotted wing drosophila (SWD). SWD is an invasive insect originating from South East Asia that threatens soft fruit production. Whereas other *Drosophila* species typically lay their eggs in (over‐) ripe or rotting fruit, female SWD, which have a serrated ovipositor, are capable of laying eggs in fruits that are still in the ripening process.^18^ Because these are the fruits that are typically used for human consumption, dramatic losses in the fruit sector have been observed.^19^, ^20^, ^21^ Currently, monitoring of SWD is conducted by placing cup‐style traps containing a liquid bait, such as a wine/vinegar mixture, in or around a field.^22^, ^23^ After a certain interval, typically around a week, the cup traps are collected and their contents are analysed manually by an expert who classifies and counts the caught insects one‐by‐one under a stereomicroscope. In this study, we explored the potential for computer vision‐based monitoring of SWD in sticky traps on a data set collected with a camera from a static position and a data set collected with the same camera mounted on a flying UAV. Both data sets where acquired outdoors under varying illumination conditions.

## 2 MATERIALS AND METHODS

### 2.1 Data collection

**Figure 1:**
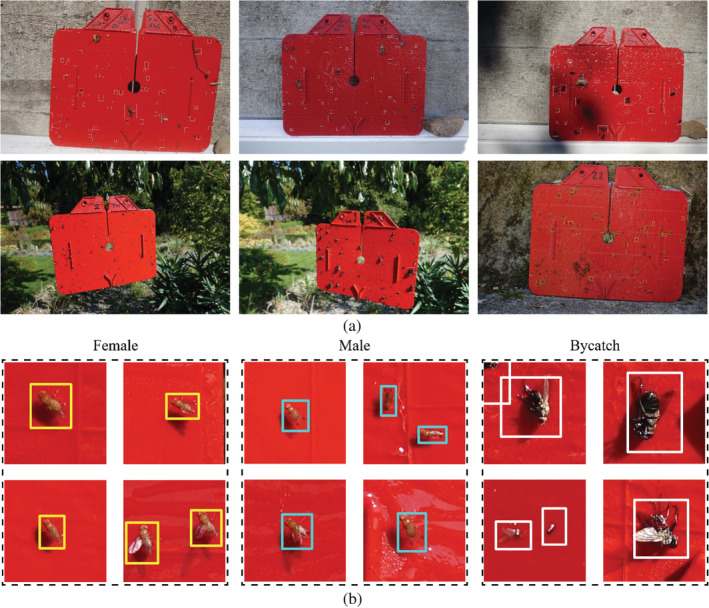
Examples of (a) training images and (b) labelled ground truth bounding boxes.

This study aimed to detect SWD in insect traps. We chose red‐coloured Rebell® sticky traps (Andermatt Biocontrol, Grossdietwil, Switzerland) because they are easily photographable. The colour red has been shown to be attractive for SWD.^24^ However, because trapping SWD *in vivo* using sticky traps remains challenging,^25^ we prepared the traps manually by placing them horizontally on the floor of an SWD breeding cage, allowing the SWD flies to land and become stuck on the sticky surface. Hereafter, traps were placed outdoors for 1 day, during which time other insect species were caught. We prepared 101 traps this way. In addition, we prepared 148 traps with only other insect species and no SWD. There were 249 traps in total. Images were collected using a Sony DSC‐RX100M4 compact camera with a 20 MP (5472 × 3648 pixels) resolution at a trap–camera distance of 50–80 cm. The images of the sticky traps were taken outside under various illumination conditions (e.g. sun‐exposed, partly or completely shadowed; see Fig. 1a), which are realistic for field situations and help to create a robust algorithm that does not only work under laboratory conditions.^26^ The images of the traps contained different complex backgrounds. An expert labelled individuals as trapped male *D. suzukii* (DSM), female *D. suzukii* (DSF), and bycatch (BC) in the images using LabelImg (https://github.com/tzutalin/labelImg) by drawing rectangular bounding boxes around them (Fig. 1b). The two sexes were discriminated under a stereomicroscope by identifying either the two black spots on the wings or the black sex combs on the first and second tarsi of the male flies, or the serrated ovipositor of the females.^27^ BC comprises many other insects that became caught in the sticky traps, for example, *Muscidae*, *Pieridae*, *Ectobius*, *Chrysopidae* and *Ensifera*, and different *Drosophila* species (there were 1531 labels for seven different *Drosophila* species). SWD are only 2–4 mm in size and therefore very small targets. In the collected images, the SWD bounding boxes were rectangular with a width to length ratio of ~ 1:1.3 and an average size of 5700 pixels, making up only 0.03% of the image.

To train, validate and test the detection model, we randomly split the 249 images as follows: 70% were used for training and 20% for validation of the model; the remaining 10% were used to test the model (Table 1). Basing the three splits on the same data set is common practice in computer vision.^28^ Moreover, it is worth noting that each image is a photograph of a unique trap. This means that the images in our test set contained only traps that had not been seen previously by the model, e.g. during training. This assures an independent test set that gives a representative indication of the model's performance. In addition to the aforementioned data set, we also collected images taken by the same camera, but mounted on an RKM4x UAV (RotorKonzept, Absteinach, Germany). Nine images of sticky traps were collected while manually hovering the UAV at approximately the same distance towards the traps as during the collection of the static data set (Table 1). These images were not used to train the model, but served to assess the potential of UAV‐based monitoring of SWD.

**Table 1:** Division of the labelled images into training, validation and test sets

	Static							UAV‐based						
	Total number of images	Number of images containing			Number of labels			Total number of images	Number of images containing			Number of labels		
		DSM	DSF	BC	DSM	DSF	BC		DSM	DSF	BC	DSM	DSF	BC
Training	173	71	61	132	1787	1795	11 715	–	–	–	–	–	–	–
Validation	50	18	9	45	165	147	3573	–	–	–	–	–	–	–
Test	26	12	13	15	444	415	1158	9	9	9	2	95	49	19

DSM, male *Drosophila suzukii*; DSF, female *D. suzukii*; BC, bycatch.

### 2.2 Model architecture and training

The detection model employed in this study corresponds to the deep CNN presented in Kellenberger *et al*.^13^ In the original setting, it was used to detect mammals in UAV images. Although SWDs are significantly smaller, the close‐up perspective from where they were photographed results in similar pixel areas covered by the flies, which allows use of the same underlying model. In detail, the model is based around a ResNet‐18,^29^ which originally yields only a single classification output per image. However, the detection setting in this work required the model to yield spatial prediction outputs. Consequently, the standard ResNet‐18 model was converted to predict a regular grid over a trap image with a down‐sampled resolution as follows: the last layers of the model (global average pooling and 1000‐way fully‐connected) were replaced with 2 one‐by‐one convolutional layers, rectified linear unit nonlinearities, dropout^30^ with 50% probability, and a final SoftMax activation. These map the 512‐dimensional feature vectors of the base ResNet output (i.e. the last residual block) to an intermediate tensor of 1024 dimensions and finally to the three output classes (background, female and male). This also meant that the model was fully convolutional and retained spatial predictions.

Training the model consisted of multiple passes over the full‐sized training set images. For every training image, 16 patches of 512 × 512 pixels were cropped at random positions from each full‐sized training set image and then used in batches of four to train the model. To artificially enlarge the training set and expose the model to different acquisition conditions, data augmentation was employed in the form of random horizontal and vertical flips as well as multiples of 90°‐stop rotations of the images. The same principles were used to tune the hyperparameters using the validation set. The Adam optimiser^31^ was used to tune the model parameters, with a learning rate of 10^−5^ for the first 50 epochs, and 10^−6^ for the remaining 250.

**Figure 2:**
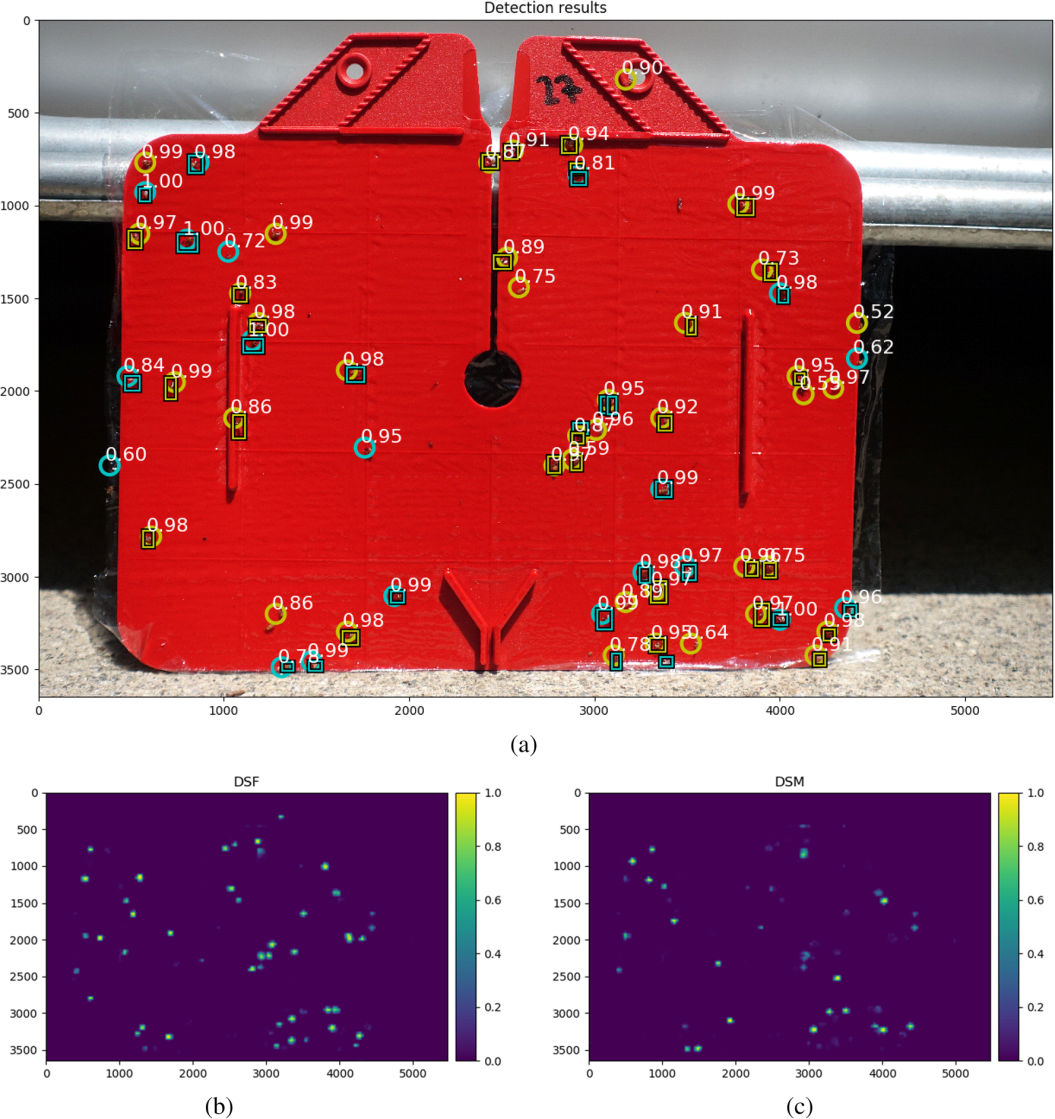
(a) Detections (circles) and their corresponding confidence values in white of female *Drosophila suzukii* (DSF; yellow) and male *D. suzukii* (DSM; blue) on an exemplary image of a sticky trap. Blue and yellow rectangles indicate the ground truth labels of DSF and DSM, respectively. Below: class probability maps of the same image as in (a) of the model for DSF (b) and DSM (c). Numbers on the *x*‐ and *y*‐axes indicate the pixel‐coordinates.

For predictions during test time, the trained model was slid over the full‐sized images as follows: images were split into a regular grid of 512 × 512 pixels; each patch was evaluated separately and final predictions were stitched back together to the full extent of the image. Because the model predicts flies on a regular grid, it is possible that an individual is recognised twice, especially if it lies on the border between multiple prediction grid cells. To reduce multiple detections of the same individual, class‐agnostic non‐maximum suppression was applied, in which only the most confident predictions were retained and others in a 3 × 3 neighbourhood with lower confidence score were discarded. The result is a down‐sampled class probability map with each position indicating the likelihood of containing either a female or male SWD, or background (Fig. 2). The model was implemented using the open source framework PyTorch (https://pytorch.org/).

### 2.3 Model evaluation

Our detection model does not predict bounding boxes, instead, it provides an image coordinate combined with a confidence value for the predicted class. Therefore, correct detections are not determined by the intersection over union between ground truth and predicted bounding box, as is commonly done to evaluate the correctness of object detections. Instead, we considered the distance in pixels between the location of the prediction and the centre of the ground truth bounding box, as in Kellenberger *et al*.^13^ Predictions were considered potentially correct if they fell within a threshold of 50 pixels from the centroid of the closest ground truth bounding box. If the predicted class was equal to the class of ground truth bounding box, and if the latter had not already been identified by another prediction, it was counted as a true positive (*Tp*). When the labels were different, or if the nearest ground truth bounding box had already been identified by another prediction, the detection was considered a false positive (*Fp*). Any ground truth bounding boxes that were missed by the model, e.g. locations where no detection was made by the model within 50 pixels, were considered a false negative (*Fn*). For the evolution of the performance of the detector, we calculated precision and recall (PR) curves for all the labels in our test sets with Equations 1, 2. Moreover, to provide a single model performance metric, we calculated the area under the PR curve (AUC) as a trade‐off between precision and recall.

(1) $$\text{Precision}=\frac{\mathrm{Tp}\;}{\mathrm{Tp}+\mathrm{Fp}}$$

(2) $$\text{Recall}=\frac{\mathrm{Tp}\;}{\mathrm{Tp}+\mathrm{Fn}}$$

## 3 RESULTS

### 3.1 General results

Figure 2 shows an example of the detection results for DSF and DSM using our model for an image of one of the sticky traps from our test data set. Detections using the model were mostly at locations where actual SWD flies were present. For this specific image and for the other images in our test data set, only a small number of false detections were made by the model (e.g. at the trap borders, and on bycatch). The probability maps on which the detections were based show that the model yielded high probabilities at different locations in the image depending on the sex of the SWD (Fig. 2b,c).

**Figure 3:**
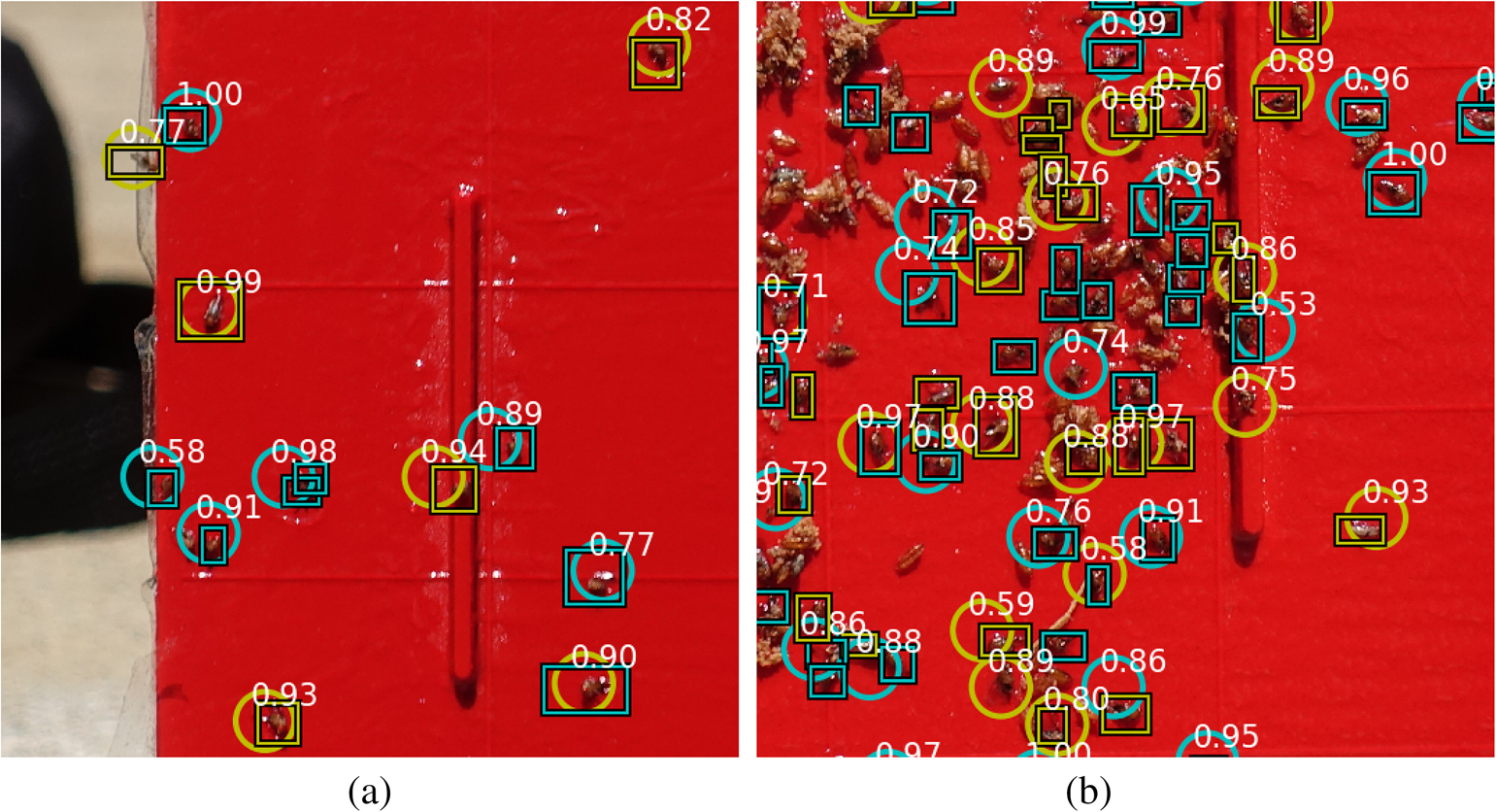
Example of the detection results of the model for the easy situation in which insects caught in the traps were separated (a), and a more complex situation in which insects caught in the traps are overlapping (b). The images were taken from a static camera position. Rectangles indicate the ground truth and circles indicate the model predictions for females (yellow) and males (blue). White numbers are the confidence levels of the predictions.

After visual inspection of the images in our test data set collected using the camera from a static position, we observed that the model was in general proficient in detecting SWD flies when they were sufficiently separated (Fig. 3a). If the flies were partially occluded or clustered together, the model was not always capable of detecting and separating them reliably. See, for example Fig. 3(b), where several DSF and DSM individuals are mixed within a cluster of bycatch of other insects that were caught in the sticky trap.

### 3.2 Results of detection in UAV images

**Figure 4:**
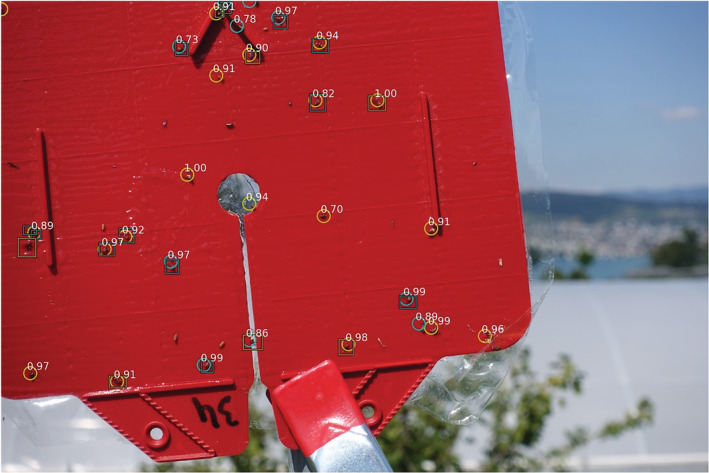
Detection of female spotted wing drosophila (SWD) (yellow) and male SWD (blue) in an image of a manually prepared sticky trap that was placed on a static position outside in the field. The image was taken by a camera carried by a flying UAV. Rectangles indicate the ground truth and circles indicate the model predictions. White numbers are the confidence levels of the predictions.

Figure 4 shows the detection results for the model on an image from the UAV‐based data set taken at approximately the same distance as the training images. Compared with the training images, the images collected by the UAV were less focused and the traps were not always captured in the centre of the image. This was due to movement of the UAV caused by the stabilisation manoeuvres, and poor positioning of the UAV towards the trap. Both can be attributed to manual operation of the UAV during image collection.

The resulting challenges in this case primarily impaired the model's accuracy in terms of sex prediction and precision (the model seems to predict a few more *Fp*). However, recall in this case was still very high, and the *Fp* are primarily bycatch and not for example, background artefacts. This is particularly noteworthy because the model had been trained on still imagery and had not been exposed to UAV‐derived acquisitions during training.

**Figure 5:**
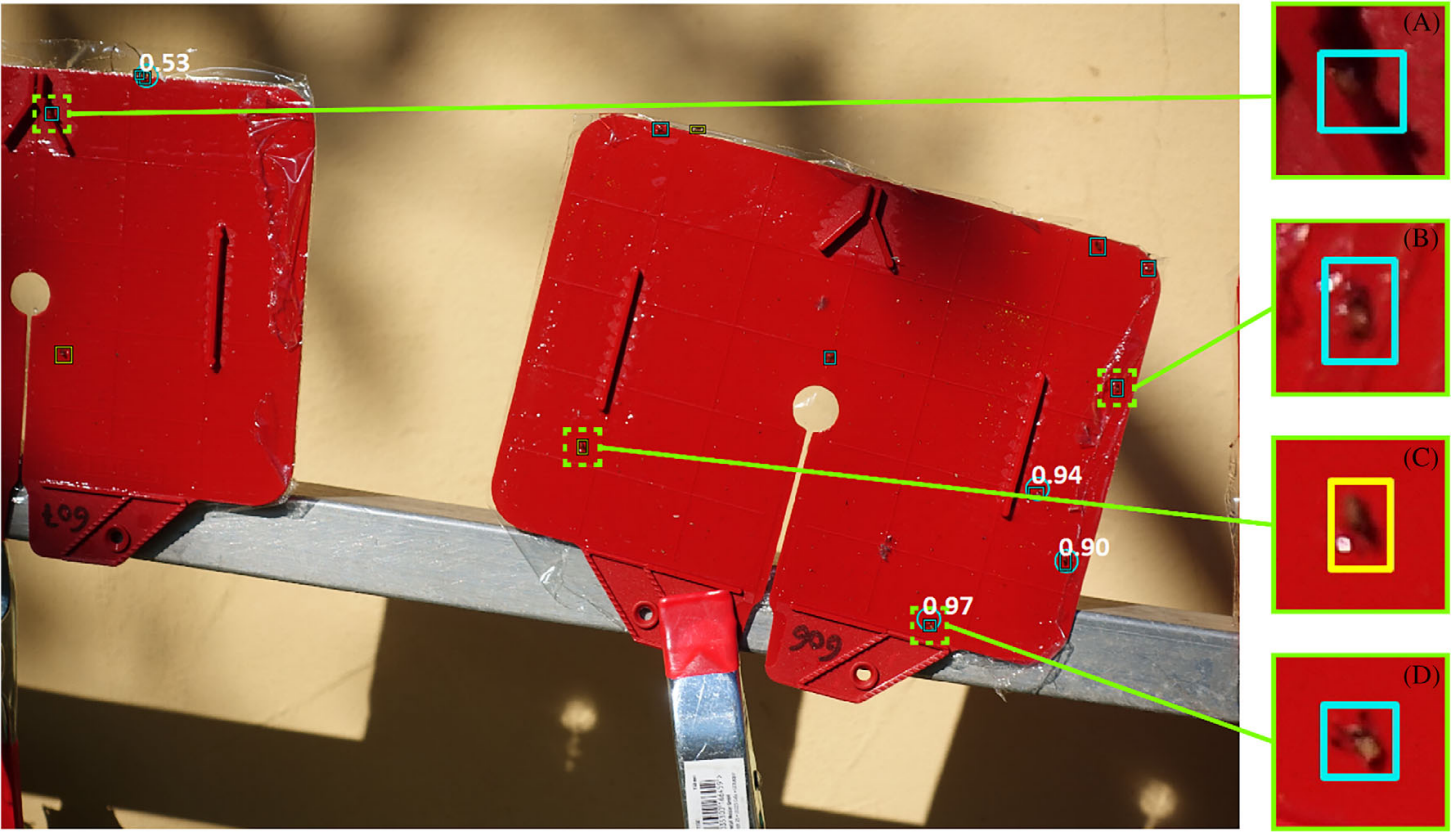
Difficulties for detection of spotted wing drosophila (SWD) in UAV‐based images caused by: (a) poor visibility due to shadows; (b) an obstructed view of SWD, in this case due to plastic foil; (c) poor image quality resulting in difficult to recognise SWD; (d) shows an easy to recognise DSM, where the black dots on the wings are clearly visible. Rectangles indicate the ground truth labels and circles are model predictions. White numbers indicate the confidence levels.

Figure 5 shows another image from the UAV‐based data set. The distance between the camera and the trap during image collection was slightly larger in this case than in the training data set. Moreover, several flies on the trap were in shaded areas (Fig. 5a) or the trapped SWD were partially occluded (Fig. 5b). As a result, only a small number of SWD present in the trap were detected by the model. Male flies that were clearly visible in the image and those for which the black dots on the wings could be recognised by the naked‐eye, such as the fly in Fig. 5(d), were consistently detected by the model.

### 3.3 Model accuracies

**Figure 6:**
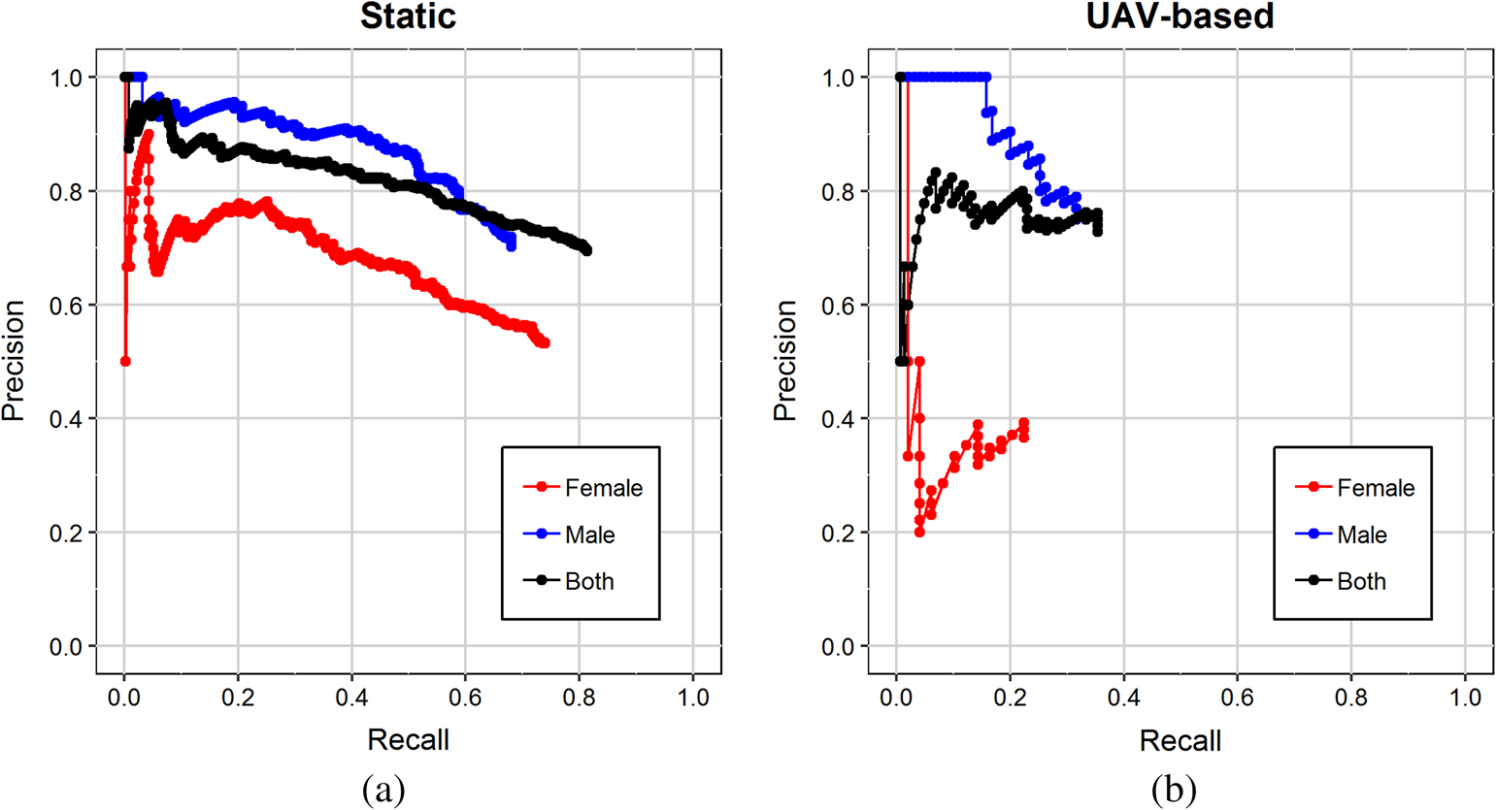
Precision‐recall curves for the data set collected from a static camera position (a) and with the same camera mounted on a flying UAV (b).

We evaluated the performance of the model using PR curves and the AUC. For the data set collected from a static position (Fig. 6a), PR curves had a recall of up to 0.73 and 0.68 for DSF and DSM, respectively. This indicates that the model was able to find more female than male SWD individuals. By contrast, male SWD could be detected with a greater precision than female SWD, as can be derived from the overall higher PR curve for males. The detection of female and male SWD resulted in AUC values of 0.506 and 0.603, respectively (Table 2). When not taking sex into consideration (‘Both’), recall of 0.82 was obtained with an AUC of 0.669. The higher precision for DSM than DSF was also found for the UAV‐based data set (Fig. 6b). However, for this data set, more males were found than females (maximum recall of 0.32 and 0.22 for male and female SWD, respectively). Compared with the static data set, the AUCs were rather low (Table 2).

**Table 2:** Area under the curve for the static data set and UAV‐based data set for female *Drosophila suzukii* (DSF), male *Drosophila suzukii* (DSM), and DSF and DSM combined (‘Both’)

Sex	Static	UAV‐based
Female (DSF)	0.506	0.086
Male (DSM)	0.603	0.284
Both	0.669	0.266

### 3.4 Fly counts

**Figure 7:**
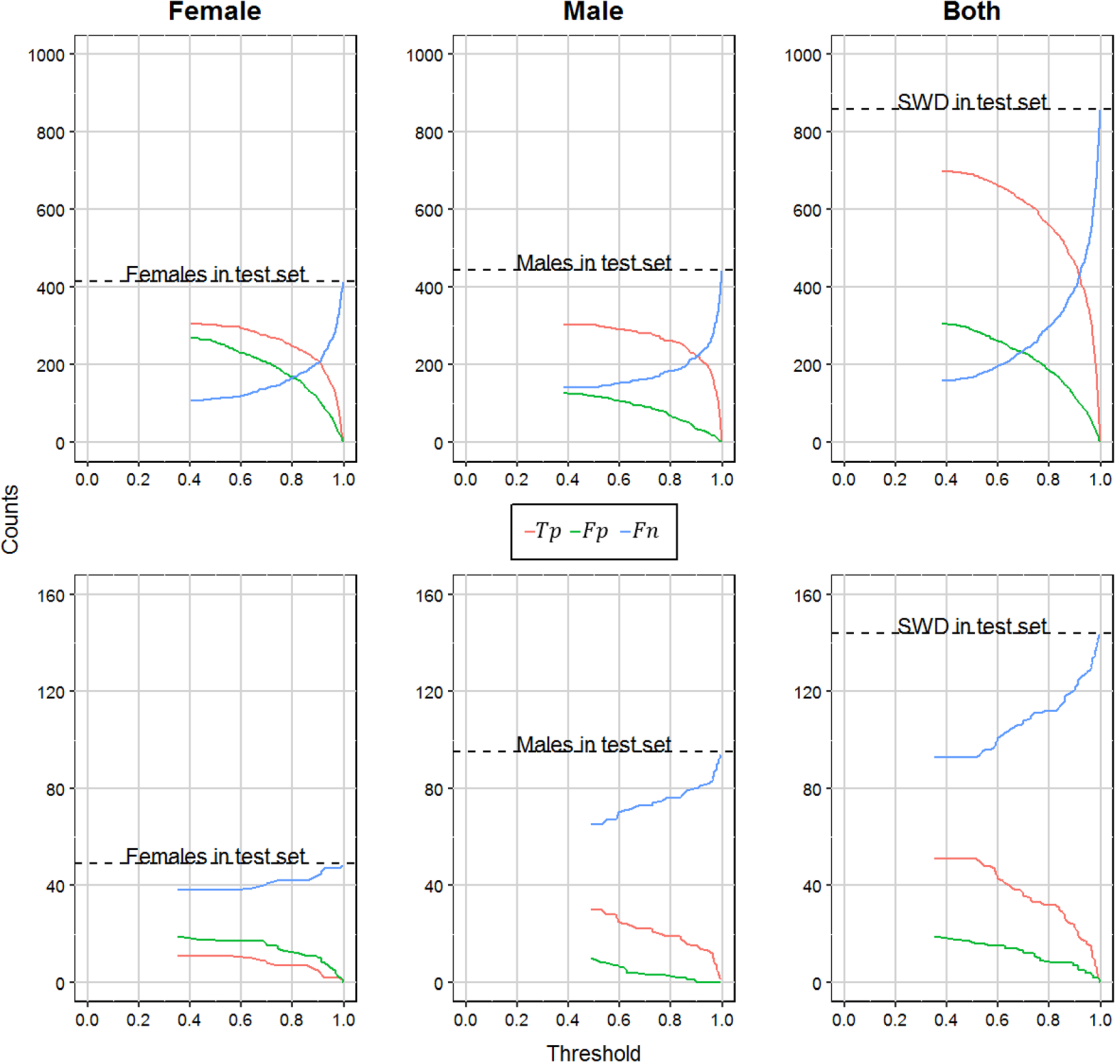
True positives (*Tp*), false positives (*Fp*), and false negatives (*Fn*) for the static data set (top row) and the data set collected with the UAV (bottom row) as a function of the detection threshold. Dashed lines indicate the total number of ground truth labels present in the test sets.

Figure 7 shows the number to true positives, false positives and false negatives for the static data set (upper row) and the data set collected with the UAV (lower row). The *Tp* and *Fp* lines follow a typical curve in which the *Tp* and *Fp* counts decrease as the detection threshold increases. By contrast, the *Fn* counts increase with an increasing detection threshold (i.e. the number of SWD detected by the model decreased when the detection threshold increased). SWD counts in the static data set were consistently higher than in the UAV data set. It is noteworthy that *Fp* counts for the male detections were consistently lower than the *Fp* counts for the female detections. For the female detections in the UAV data sets, the *Fp* counts even exceeded the *Tp* counts (Fig. 7, bottom left).

## 4 DISCUSSION

Here, we explored the possibility of detecting SWD individuals in sticky trap images using deep learning and object detection. We evaluated this for a data set of images collected with a camera from a static position and for a data set collected with the same camera mounted on a flying UAV. While doing so, we attempted to make a distinction between male and female flies. Our model showed very different predictions for the locations in the test imagery for male and female flies (Fig. 2b,c), which indicates that it learned to distinguish between the two sexes. Our results show that male SWD can be detected with higher precision than female SWD (Fig. 6a). One possible explanation for this is that male SWD are more distinctive than other *Drosophila* species in our data set, owing to the black spots on their wings (Fig. 1b). Females do not have these spots, which makes them more similar to other *Drosophila* species. Likely, this resulted in more false detections for females and therefore in lower precision for this class.

Similar results were found for the UAV‐based data set, although recall was consistently lower than for the static data set (Fig. 6), indicating that a smaller number of flies present were detected by the model. This is very likely due to the fact that the UAV‐based imagery was of lower quality than that collected from a static camera. Not only were the UAV‐based images less sharp and less well focused, but the distance and angle between the camera and the trap were also more variable. The suboptimal position of the UAV in relation to the trap was the result of manual operation during image collection. In addition to operating the UAV and taking the images, the operator had to maintain a safe distance to make sure that the UAV did not collide with the trap. In future, manual operation of the UAV could potentially be replaced by autonomously flying UAVs, which might result in a better and more stable position of the UAV towards the trap and therefore higher image quality, and thus greater precision and recall of the detections. In addition, training the model on UAV‐derived imagery directly could accustom it to the domain of the nonstationary UAV images and further improve its performance.

Monitoring pest insects using deep learning object detection methods is a niche area of computer vision and therefore assessment of model performance is also not standardised.^3^, ^32^ Moreover, the specific case of detecting a particular species of fruit fly and distinguishing its sex has not been studied until now. Our detection results were relatively low compared with other insect detection research that has been published. However, these studies used highly standardised and optimised imagery to train and test their models. For example, Sun *et al*.^9^ obtained an AUC of 0.746 for the in‐trap detection of red turpentine beetle. The controlled conditions (i.e. fixed camera–trap distance, exposure control with LED light, and standard white background) under which their training and test imagery was collected contributed to this high accuracy. We believe, that with better and more consistent image quality, the detection results for SWD can improve significantly.

Current off‐the‐shelf UAV‐based systems are not capable of collecting images of high enough quality for the detection of objects as small as the target insect species in our study. The camera systems of these UAVs typically have wide‐angle lenses and/or are focused to infinity, which complicates collecting imagery of nearby objects. Therefore, for this study, we used a custom UAV system with a higher‐grade camera that was capable of collecting suitable imagery. The downside of such a system is that it is currently more expensive, is less stable than the off‐the‐shelf systems when hovering, and requires expert knowledge to operate it.

In our data set, we observed that when the image quality was sufficient, i.e. when the black spots on the wings of male SWD were visible to the naked eye, the model was generally capable of making the correct class prediction. However, taking images in which the spots are visible is very challenging, especially when done with a camera mounted on a UAV in manual operation mode. With the current rate of advancements and developments in the field of UAVs in terms of stabilisation, flight‐autonomy and camera quality, this will be less of an issue in the near future.^33^ An alternative would be to use traps with automated fixed cameras. These systems have become available commercially and as a result of the miniaturisation of optics and electronics, costs are becoming lower. However, if spatially high resolutions are needed, the cost of fixed camera trap systems quickly become too high, as opposed to, for example, cheap traps with a mobile camera. Moreover, trap imaging with cameras mounted on ground‐based vehicles has been suggested,^34^ but the accessibility and manoeuvrability of these vehicles might be a limitation. UAV‐based approaches could be seen as a complementary alternative. In situations in which camera‐based traps cannot be left in the field, flexibility in camera type is required (e.g. multi‐spectral and thermal),^35^ or in case of large area coverage, UAV‐based systems could result in lower costs as overall fewer cameras would be required. Although operational UAV‐based monitoring for pest detection is still to be devised completely, our results demonstrate the proof‐of‐concept that there is potential for these platforms to perform the task.

## 5 CONCLUSIONS

In this paper, we demonstrated that it is possible to detect *D. suzukii* in images of sticky traps using object detection and deep learning. Using a deep CNN, we were able to detect flies with an AUC of 0.506 and 0.603 for female and male flies, respectively, for an image data set collected from a static camera position. This not only shows that the specific fruit fly species in our study can be detected, but also that the sexes can be distinguished reliably. Moreover, we have shown that *D. suzukii* detection is possible in imagery collected by UAVs, highlighting the potential of UAVs for monitoring, which can be valuable for IPM. This paper demonstrated the specific case for *D. suzukii* monitoring as a proof‐of‐concept for pest detection using UAVs. In future, the same strategy could also be applied for the detection of other pests.
